# Latexin sensitizes leukemogenic cells to gamma-irradiation-induced cell-cycle arrest and cell death through Rps3 pathway

**DOI:** 10.1038/cddis.2014.443

**Published:** 2014-10-23

**Authors:** Y You, R Wen, R Pathak, A Li, W Li, D St Clair, M Hauer-Jensen, D Zhou, Y Liang

**Affiliations:** 1 Department of Internal Medicine, University of Kentucky, Lexington, KY 40536, USA; 2 Division of Radiation Health, Department of Pharmaceutical Sciences, University of Arkansas for Medical Sciences, Little Rock, AR, USA; 3 Department of Pathology, Harvard Medical School, Boston, MA 02115, USA; 4 Gratuate Center for Toxicology, University of Kentucky, Lexington, KY 40536, USA

## Abstract

Leukemia is a leading cause of cancer death. Recently, the latexin (Lxn) gene was identified as a potential tumor suppressor in several types of solid tumors and lymphoma, and Lxn expression was found to be absent or downregulated in leukemic cells. Whether Lxn functions as a tumor suppressor in leukemia and what molecular and cellular mechanisms are involved are unknown. In this study, the myeloid leukemogenic FDC-P1 cell line was used as a model system and Lxn was ectopically expressed in these cells. Using the protein pull-down assay and mass spectrometry, ribosomal protein subunit 3 (Rps3) was identified as a novel Lxn binding protein. Ectopic expression of Lxn inhibited FDC-P1 growth *in vitro*. More surprisingly, Lxn enhanced gamma irradiation-induced DNA damages and induced cell-cycle arrest and massive necrosis, leading to depletion of FDC-P1 cells. Mechanistically, Lxn inhibited the nuclear translocation of Rps3 upon radiation, resulting in abnormal mitotic spindle formation and chromosome instability. Rps3 knockdown increased the radiation sensitivity of FDC-P1, confirming that the mechanism of action of Lxn is mediated by Rps3 pathway. Moreover, Lxn enhanced the cytotoxicity of chemotherapeutic agent, VP-16, on FDC-P1 cells. Our study suggests that Lxn itself not only suppresses leukemic cell growth but also potentiates the cytotoxic effect of radio- and chemotherapy on cancer cells. Lxn could be a novel molecular target that improves the efficacy of anti-cancer therapy.

Leukemia is one of the most common cancers and one of the leading causes of cancer death. In the United States, an estimated 52 380 new cases of leukemia will be diagnosed in 2014, and approximately 24 090 patients will die of this disease (NCI Stat Fact Sheets: http://seer.cancer.gov/statfacts/html/leuks.html). Leukemia can be treated by chemotherapy, radiation therapy and bone marrow transplantation. Radiation and most chemotherapeutic reagents induce apoptosis in tumor cells, resulting in tumor regression. However, some cancer cells are resistant to therapy-induced apoptosis, leading to treatment failure and cancer relapse.^1,2^ There is an urgent need for discovery of novel molecular targets that can abrogate death resistance in leukemic cells and enhance the cytotoxic efficacy of anti-cancer therapies.

Latexin (Lxn) could be an interesting candidate for this purpose. Lxn is a novel regulator of hematopoietic stem cell function and homeostatic hematopoiesis.^3^ It is downregulated in several types of solid and liquid tumors, and overexpression of Lxn inhibits tumor cell growth.^4, 5, 6, 7, 8^ In lymphoma, the tumor suppressor function of Lxn is mediated through Bcl-2-induced apoptosis.^5^ Moreover, *Lxn* was reported to be a TNF-responsive gene in human papillovirus-infected keratinocytes, suggesting that it may contribute to TNF-induced suppression of cervical cancer.^9^ Lxn is also implicated in inflammation because it is highly enriched in mast cells and can be upregulated by lipopolysaccharide.^10,11^ In addition, Lxn regulates the interaction of hematopoietic stem/progenitor cells to stroma through altering the abundance of cell adhesion molecules.^12^ The only known Lxn binding protein is carboxypeptidase A (CPA), and it inhibits CPA activity, indicating that Lxn might be involved in protein degradation and metabolism.^10,13, 14, 15^ However, we have already shown that the tumor suppressor function of Lxn is not through the canonical CPA pathway in lymphoma cells.^5^

Currently, the mechanism of action of Lxn in normal and malignant conditions remains unknown, and no reports have been made as to other proteins that could bind to Lxn. In this study, we aimed to discover novel Lxn binding proteins, and evaluate whether Lxn could enhance the cytotoxic effect of radiation and chemotherapeutic agent on leukemic cells. We used myeloid leukemogenic progenitor cell line FDC-P1 as a model system and ectopically expressed Lxn in these cells.^16, 17, 18^ Using a protein pull-down assay and mass spectrometry (MS), we identified ribosomal protein subunit 3 (Rps3) as a novel Lxn binding protein. We then examined the response of Lxn-overexpressing FDC-P1 cells to gamma-irradiation and found that Lxn sensitizes these cells to radiation-induced cell death and inhibits tumor cell growth. FDC-P1 cells with ectopic Lxn expression demonstrate more DNA double-strand breaks (DSBs) upon irradiation, which triggers a dramatic G2/M arrest and blocks G1- and S-phase entry. The abnormal cell-cycle progression results in massive necrosis and depletion of Lxn-overexpressing cells. Mechanistically, the increased level of Lxn reduces nuclear translocation of Rps3 upon radiation, which causes abnormal mitotic spindle formation and chromosome instability. Moreover, Rps3 knockdown increases the radiation sensitivity of FDC-P1 cells, confirming that Rps3 is involved in Lxn-mediated radiation response. In addition, Lxn enhances cytotoxicity of chemotherapeutic agent, VP-16, on FDC-P1 cells. This study, for the first time, unravels a mechanistic role of Lxn as a tumor suppressor *via* a previously unknown Rps3 pathway. Lxn could be a novel molecular target that improves the efficacy of anti-cancer therapy.

## Results

### RPS3 is a novel Lxn binding protein

**Figure 1:**
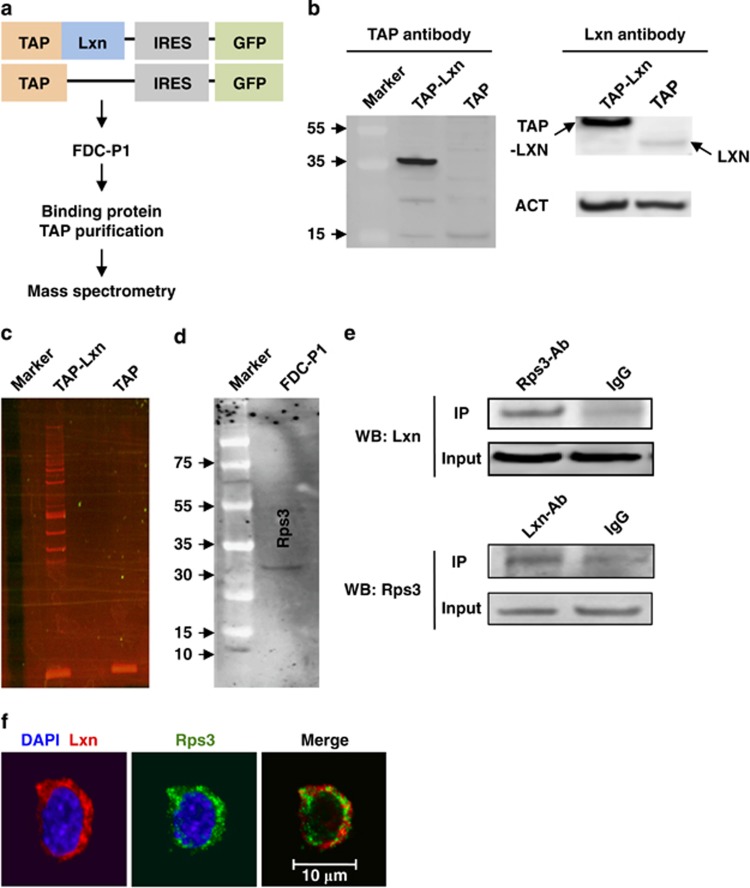
Rps3 is a novel Lxn binding protein. (**a**) Experimental scheme for isolation of Lxn binding protein. Full-length mouse Lxn was fused in-frame to an N-terminal tandem affinity purification (TAP) tag and sub-cloned into Sf*β*9.1-GFP retroviral vector to create the TAP-Lxn vector. A plasmid carrying the TAP tag was used as a control (TAP). FDC-P1 cells were transfected with the TAP-Lxn or TAP retroviral supernatant, and GFP+ cells were sorted by flow cytometry to establish GFP+ stable cell line. Whole-cell lysates from TAP-Lxn or TAP FDC-P1 cells were purified with the InterPlay Mammalian TAP purification kit and the differentially expressed bands were subjected to mass spectrometry. (**b**) Detection of TAP-Lxn or TAP protein expression in stable GFP+FDC-P1 by antibodies against TAP tag (left panel) and Lxn (right panel), respectively. (**c**) Proteins differentially present in FDC-P1 cells transfected with TAP-Lxn or TAP vectors. (**d**) Rps3 protein is expressed in FDC-P1 cells. Western blot was performed with anti-Rps3 antibody. (**e**) Co-immunoprecipitation confirms the binding of Lxn and Rps3. Whole-cell lysate from FDC-P1 cells was incubated with Rps3 antibody or IgG control, and the precipitated proteins were probed with anti-Lxn antibody in western blot (WB:Lxn) (top panel). The binding was confirmed by the reciprocal way in which the protein complex was precipitated by Lxn antibody and then western blotted with Rps3 antibody (WB: Rps3) (bottom panel). (**f**) Lxn and Rps3 are colocalized in the cytoplasm of FDC-P1 cells. FDC-P1 cells were fixed and stained with immunofluorescent probes for Lxn and Rps3 proteins. Lxn was detected by polyclonal antibody and rhodamine-conjugated goat-anti-chicken secondary antibody; Rps3 was detected by polyclonal antibody and FITC-conjugated goat anti-rabbit secondary antibody. Scale bar represents 10 *μ*m

Lxn is the only known CPA inhibitor in mammals; it binds to CPA4 in humans and CPA1 in mouse.^10,14^ We have previously shown that the mechanism of action of Lxn is not through inhibition of CPA in lymphoma cells.^5^ Thus, we used the tandem affinity purification (TAP) method in combination with MS to screen novel Lxn binding proteins in FDC-P1 cells, a murine leukemogenic cell line, that can induce myeloid leukemia *in vivo* (Figure 1a).^17,18^ We first detected the expression of TAP-Lxn fusion protein with TAP antibody, and found that the fusion protein was expressed only in FDC-P1 cells transduced with TAP-Lxn vector but not with TAP vector (Figure 1b, left panel). By using Lxn antibody, we confirmed overexpression of Lxn protein in TAP-Lxn-transduced cell compared with a very low level of endogenous Lxn in FDC-P1 cells (Figure 1b, right panel). It should be noted that TAP tag itself has ~7.7 kDa molecular weight, thus TAP-Lxn fusion protein is around 35 kDa. We next detected proteins differentially present in TAP-Lxn cells and extracted them for LC-MS analysis (Figure 1c). Eleven proteins with significant score were identified, including Lxn itself (Supplementary Table 1). Among these interacting proteins, Rps3 was of particular interest because it has important extra-ribosomal role in DNA damage response and in the regulation of p53 degradation and NF-κB signaling pathways.^19, 20, 21, 22, 23, 24^

We confirmed the expression of Rps3 protein in FDC-P1 cell by western blot (Figure 1d). Protein complex immunoprecipitation (Co-IP) further validates the binding of Lxn and Rps3. The result showed that Lxn was precipitated by Rps3 antibody, and reciprocally, Rps3 was precipitated by Rps3 antibody (Figure 1e, top and bottom panel, respectively). Moreover, immunocytochemical staining result showed that Rps3 and Lxn were colocalized in the cytoplasm (Figure 1f). Therefore, Rps3 is a novel Lxn binding protein and both are localized in the cytoplasm of FDC-P1 cell in non-stressed condition.

### Lxn inhibits FDC-P1 cell growth in normal and stress conditions

**Figure 2:**
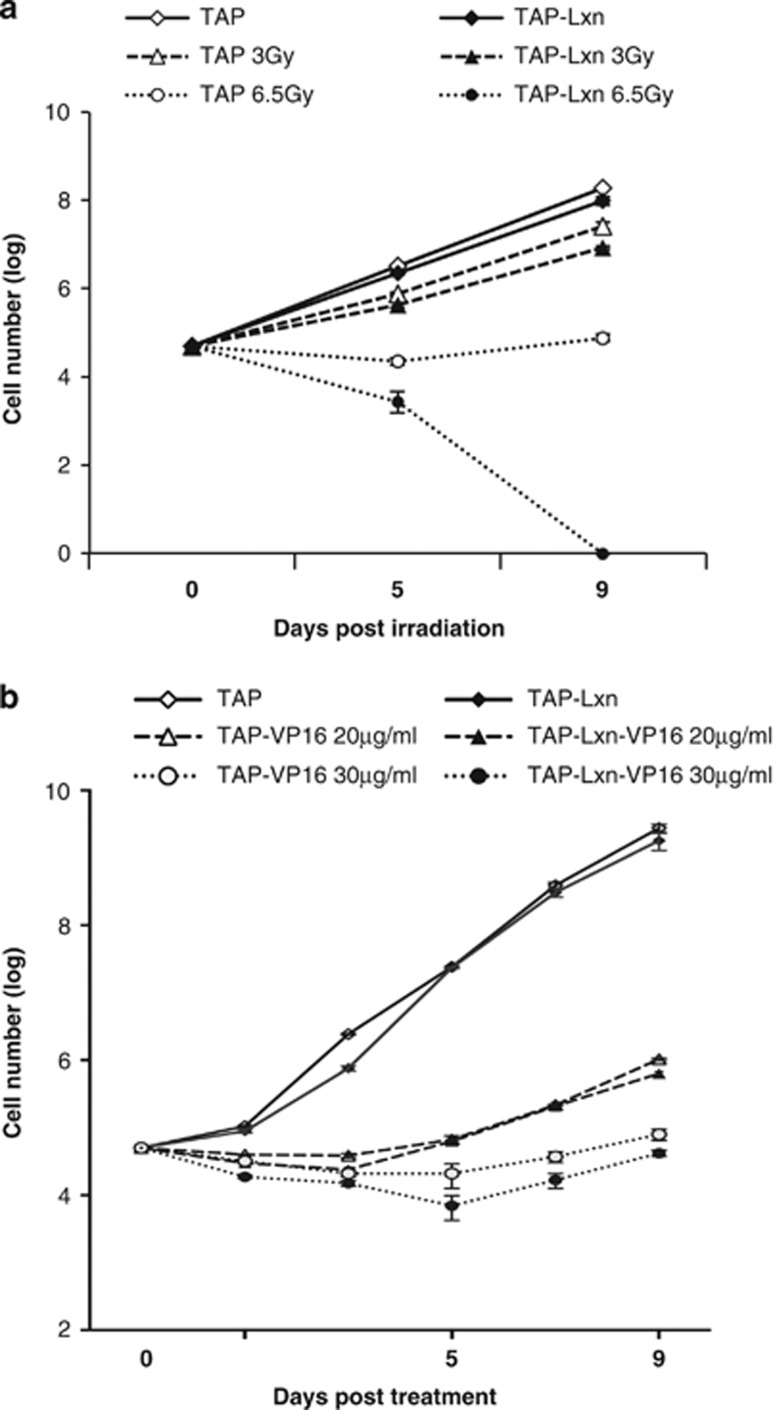
Lxn potentiates cytotoxicity of radiation and chemotherapy drug. (**a**) Lxn increases radiation sensitivity of FDC-P1 cells and inhibits their growth in a dose-dependent manner. FDC-P1 cells transduced with TAP-Lxn or TAP vector were treated with different doses of radiation (3 or 6.5 Gy). (**b**) Lxn enhances cytotoxicity of chemotherapy drug. FDC-P1 cells transduced with TAP-Lxn or TAP vector were treated with different doses of VP-16 (20 or 30 *μ*g/ml). Cell growth was measured by trypan blue cell count at indicated time points for 9 days. The cell number was represented by log scale. Three independent experiments were performed and the data were presented by the average±standard deviation (S.D.). *P*-values at each time point were indicated in the text

Rps3 has an important role in DNA damage and repair.^21,23,25, 26, 27, 28, 29^ Lxn is a negative regulator of hematopoietic stem and progenitor cell population size.^3,12,30^ We asked whether Lxn overexpression could affect the growth of FDC-P1 cells in normal and stress conditions. FDC-P1 cells (TAP-Lxn or TAP transfected) were irradiated with different doses of gamma-irradiation (3 and 6.5 Gy), and the cell number was monitored over a period of 9 days of culture after radiation. In the untreated condition, overexpression of Lxn resulted in a 2-fold decrease in the number of FDC-P1 cells (Figure 2; *P*=0.007 at day 5 and *P*=0.006 at day 9) although the same number of cells was seeded into the culture at day 0 in both groups. Radiation stress dramatically reduced the cell growth and demonstrated a dose-dependent response. TAP-Lxn cells showed a 2-fold decrease in number at day 5 after 3 Gy irradiation (*P*=0.005), and it further decreased to 8-fold with the dose of 6.5 Gy (*P*=0.009). Lxn-mediated growth suppression was further amplified for the following days of culture and by day 9 nearly 3-fold fewer cells were present in TAP-Lxn group than in control group (*P*=0.03) at 3 Gy. More surprisingly, TAP-Lxn cells were completely depleted by 6.5 Gy irradiation at day 9, whereas a certain number of control cells were recovered and started to grow.

We next determined cellular response of TAP-Lxn and TAP-transduced FDC-P1 cells to another DNA damage agent, VP-16. VP-16 is an anti-cancer chemotherapy drug and classified as topoisomerase II inhibitor. Figure 2b shows that VP-16 dramatically inhibited FDC-P1 growth and had a dose-dependent effect in the reduction of cell quantity. At day 9, the number of TAP-Lxn cells was nearly 2-fold less than control group at the dose of 30 *μ*g/ml (*P*=0.005). Altogether, Lxn inhibits myeloid leukemic cell growth and sensitizes them to radiation and chemotherapy-induced cytotoxicity. Since radiation exerts more profound effect on Lxn-overexpressing cells, the following mechanistic study will focus on this condition. It is plausible that the similar mechanism may be applied to Lxn-mediated chemo-sensitivity.

### Lxn causes chromosomal instability and increases radiation-induced DNA damages

Gamma irradiation causes DNA DSBs.^31^ We asked whether the increased level of DNA damages and/or less efficient repair results in the radio-sensitivity of Lxn-overexpressing FDC-P1 cells. We measured the dynamic formation of phosphorylated H2A.X (*γ*-H2A.X), a marker for DNA DSB,^32,33^ in FDC-P1 cells after a low dose of 2 Gy irradiation. Figure 3a shows that the number of *γ*-H2A.X foci at a single cell level was significantly increased at early time point (1 h) post irradiation in TAP and TAP-Lxn cells but no significant difference was observed between two groups. However, TAP-Lxn cells demonstrated a significantly higher number of *γ*-H2A.X foci than control cells from 3 h (Figures 3a and b) to 24 h post irradiation. These data suggest that Lxn may be involved in DSB repair, and increased level of Lxn attenuates the repair leading to accumulation of DNA damage.

**Figure 3:**
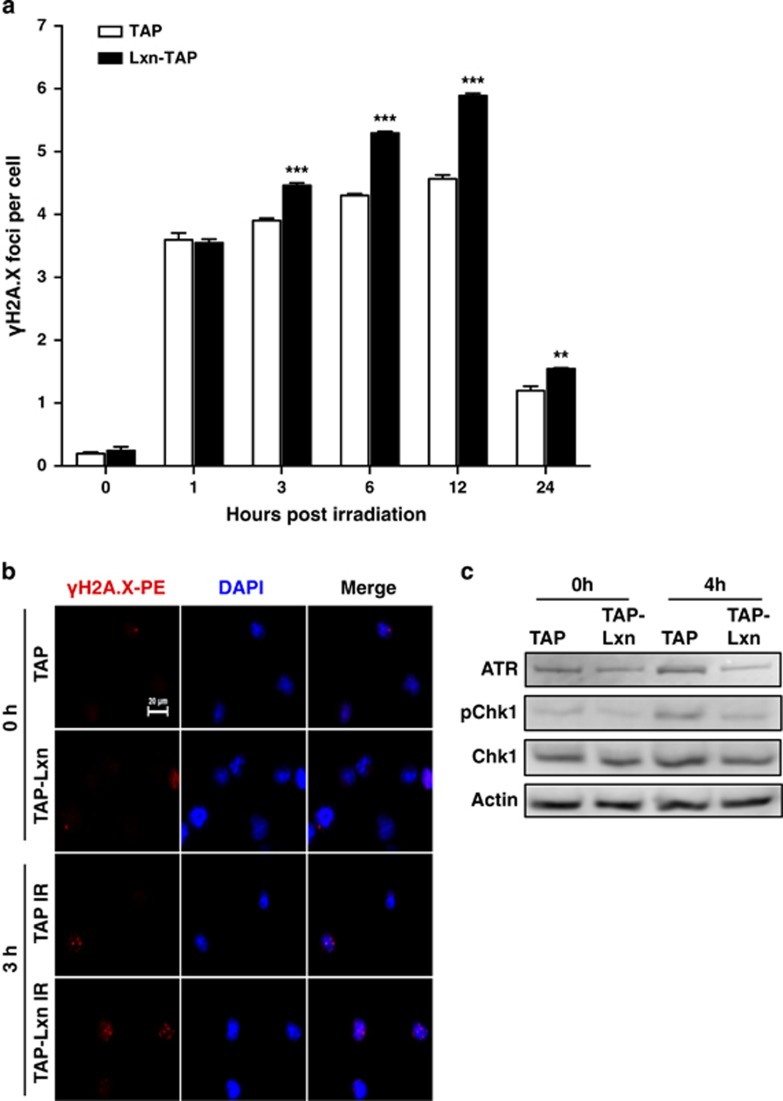
Lxn increases radiation-induced DNA damages and inhibits DNA damage response checkpoints. (**a**) Lxn-overexpressing FDC-P1 cells had more radiation-induced DNA DSB). FDC-P1 cells transfected with TAP-Lxn or TAP control vectors were irradiated at a dose of 6.5 Gy and DSBs were detected by *γ*-H2A.X-PE staining for the formation of *γ*-H2A.X foci during 24 h post irradiation. Y axis represents the average number of *γ*-H2A.X foci per cell out of 60–80 cells. **P<0.01 and ***P<0.001. (**b**) Representative immnunofluorescent imaging of *γ*-H2A.X foci in FDC-P1 cells at 3 h post irradiation. (**c**) Lxn inhibits the activation of ATR-Chk1 pathway. Expression of ATR and native and phosphorylated Chk1 (pChk1) was detected by western blot in TAP-Lxn and TAP cells at 4 h post 6.5 Gy irradiation

**Figure 4:**
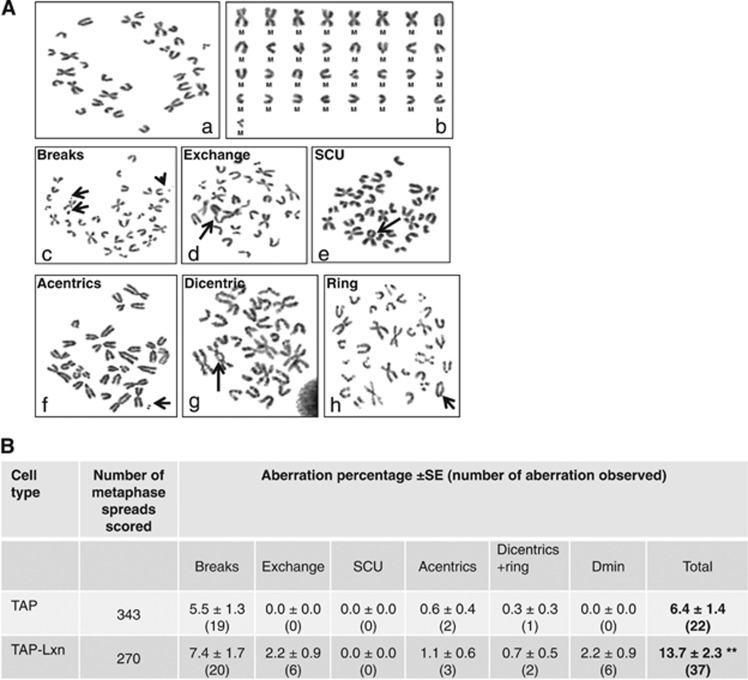
Lxn increases chromosomal instability. (**A**) Representative photomicrograph showing different structural chromosomal aberrations. Arrows indicate (a) normal metaphase spread with 33 chromosomes, (b) karyotype of the normal metaphase showing seven metacentric chromosomes, (c) chromatid-type breaks, (d) chromatid-type exchange, (e) sister chromatid union, (f) acentric fragment, (g) dicentric chromosome and (h) ring chromosome. (**B**) Lxn increases chromosomal aberrations. Aberration percentage±S.E. (total number of aberration observed) detected in TAP control and TAP-Lxn FDC-P1 cells. Standard errors on the aberration percentage were calculated by √*a/A*, where *a* is the number under consideration and *A* is the total number of cells analyzed. The abbreviations used for chromosomal aberrations are according to international nomenclature.^47^ Z-test was used for group comparison.^46^ **P<0.01

We next examined expression of proteins involved in DNA damage response, including ATM, ATR, phosphorylated Chk2 and Chk1, and p53. Although ATM-Chk2 pathway has a predominant role in cellular response to radiation, we did not detect any significant difference in the expression of ATM and phosphorylated Chk2 between TAP-Lxn and TAP cells at 4 h post 6.5 Gy irradiation (data not shown). Instead, we found that the protein level of ATR and phosphorylated Chk1 was lower in TAP-Lxn cells than in control cells in non-irradiated condition, and upon radiation, the activation of ATR-Chk1 axis was significantly inhibited in TAP-Lxn cells (Figure 3c). This result suggests that increased level of Lxn could inhibit ATR-Chk1 pathway, resulting in a deficient surveillance mechanism. These changes could cause chromosomal instability in non-stress condition and accumulation of more DNA DSBs upon radiation. To confirm the chromosomal instability in non-stress condition, we performed the cytogenetic study to quantify chromosomal aberrations. Figure 4A is the photomicrograph showing different structural aberrations in FDC-P1 cell. Figure 4B summarizes the quantification of each type of aberrations out of total ~300 cells, and shows that TAP-Lxn cells had a significant higher percentage of aberrant chromosomes than control cells (13.7±2.3 *versus* 6.4±1.4, *P*<0.01).

In addition, we found that FDC-P1 cells did not express another critical checkpoint protein, p53, which was consistent with the literature (data not shown).^34^ Altogether, Lxn impairs DNA repair capacity and suppresses DNA damage response, leading to accumulation of unrepaired DNA damages in Lxn-overexpressing cells. These cells will probably bypass cell-cycle checkpoint due to lack of p53, accumulate in the later phase of cell cycle, and ultimately undergo massive cell death.

### Lxn alters cell-cycle progression and induces cell-cycle arrest

**Figure 5:**
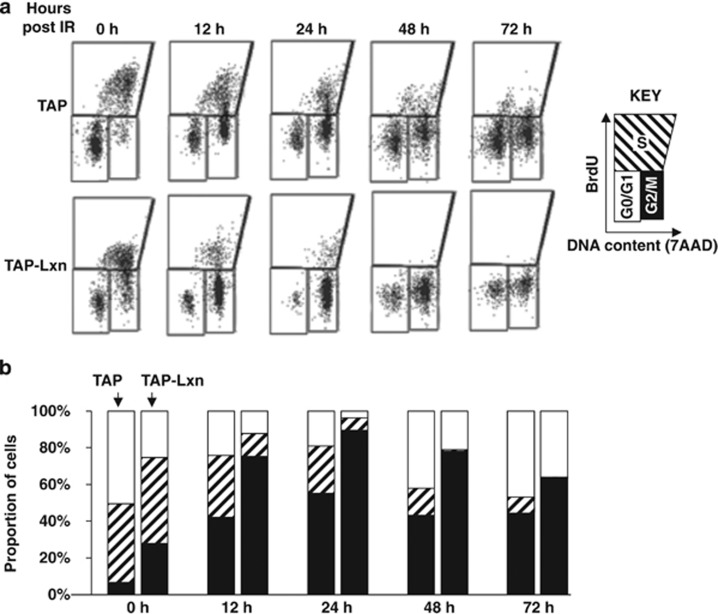
Lxn sensitizes FDC-P1 cells to radiation-induced cell-cycle arrest. Lxn enhances the radiation-induced cell-cycle arrest at G2/M phase and blocks the entry of G1 and S phases. TAP-Lxn or TAP-transfected FDC-P1 cells were treated with 6.5 Gy irradiation and the cell-cycle progression was analyzed by BrdU and 7AAD staining and flow cytometry at indicated time points. The representative flow cytometry profile out of three independent experiments was shown in (**a**) and quantification of different phases of the cell cycle (G0/G1, S and G2/M phases) was shown in (**b**)

To determine the role of Lxn in DNA damage response, we studied the cell-cycle progression with BrdU and 7AAD using flow cytometry (Figure 5a). In the non-irradiated condition (0 h), Lxn altered the cell-cycle distribution as follows (Figure 5b): the fraction of G0/G1 population in TAP-Lxn cells was half of TAP control cells (25 *versus* 50%), and correspondingly nearly 4-fold more TAP-Lxn cells were present in G2/M phase (27 *versus* 7%). After irradiation, the fraction of cells in the G2/M phase significantly increased at all time points but the increase was even higher in TAP-Lxn group than in control. At 24 h, nearly 90% of TAP-Lxn cells were present in the G2/M phase whereas around 50% of TAP control cells were in this phase. The increased population of G2/M-phase cells corresponded well with the reduced population of G0/G1 cells, suggesting that Lxn prevents cells from returning to the G0/G1 phase, thus blocking normal cell-cycle progression. As a result, TAP-Lxn cells demonstrated a dramatic decrease in the fraction of S phase after irradiation. At 24 h, only 6.2% of cells held at S phase compared with control (23.5%), and a nearly complete depletion of S population in TAP-Lxn cells at 48 and 72 h. Thus, radiation-induced cell-cycle arrest and depletion of proliferating cells may contribute to the elimination of Lxn-overexpressing cells (see Figure 2a).

### Lxn potentiates radiation-induced cell death

**Figure 6:**
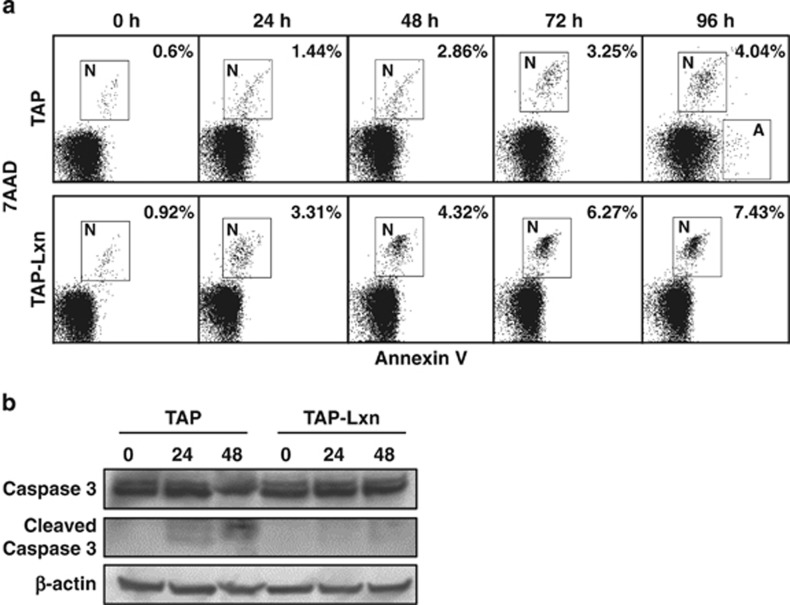
Lxn enhances radiation-induced necrosis. (**a**) Lxn-overexpressing FDC-P1 cells undergo necrosis in response to radiation. TAP-Lxn or TAP-transfected FDC-P1 cells were treated with 6.5 Gy radiation and cell death was determined by Annexin V and 7AAD staining and flow-cytometry analysis. Annexin V-positive and 7AAD-negative cells are apoptotic (labeled as A) and cells positive for both makers are necrotic (labeled as N). Representative flow-cytometry profiles were shown and percentages of apoptotic and necrotic cells at indicated time points post irradiation were labeled. (**b**) Lxn-overexpressing FDC-P1 cells lack the activation of caspase 3 upon irradiation. Western blot was performed with the antibody against caspase 3 to detect intact and active (cleaved) forms of caspase 3. *β*-Actin was used as the control

To determine whether the abnormal cell-cycle progression and accumulation of DNA damages in Lxn-overexpressing FDC-P1 cells could lead to cell death, we used Annexin V and 7-AAD staining and flow cytometry to determine apoptosis and necrosis. Upon 6.5 Gy irradiation, TAP-Lxn cells directly underwent necrosis without initiating early apoptotic response, whereas a proportion of control cells was apoptotic (Figure 6a). At all detected time points, nearly 2-fold more TAP-Lxn cells were necrotic than control cells, which may explain the rapid clearance of these cells during 9 days of cell culture (see Figure 2a). Such different cell death response was also confirmed at the molecular level. Caspase-3, a primary executioner of apoptosis, was not activated in TAP-Lxn cells but its active form was detected in control cells from 24 h post irradiation (Figure 6b). These data suggest that Lxn is involved in the regulation of cell death response.

### Lxn inhibits nuclear translocation of Rps3 upon irradiation and induces abnormal mitotic spindle formation

**Figure 7:**
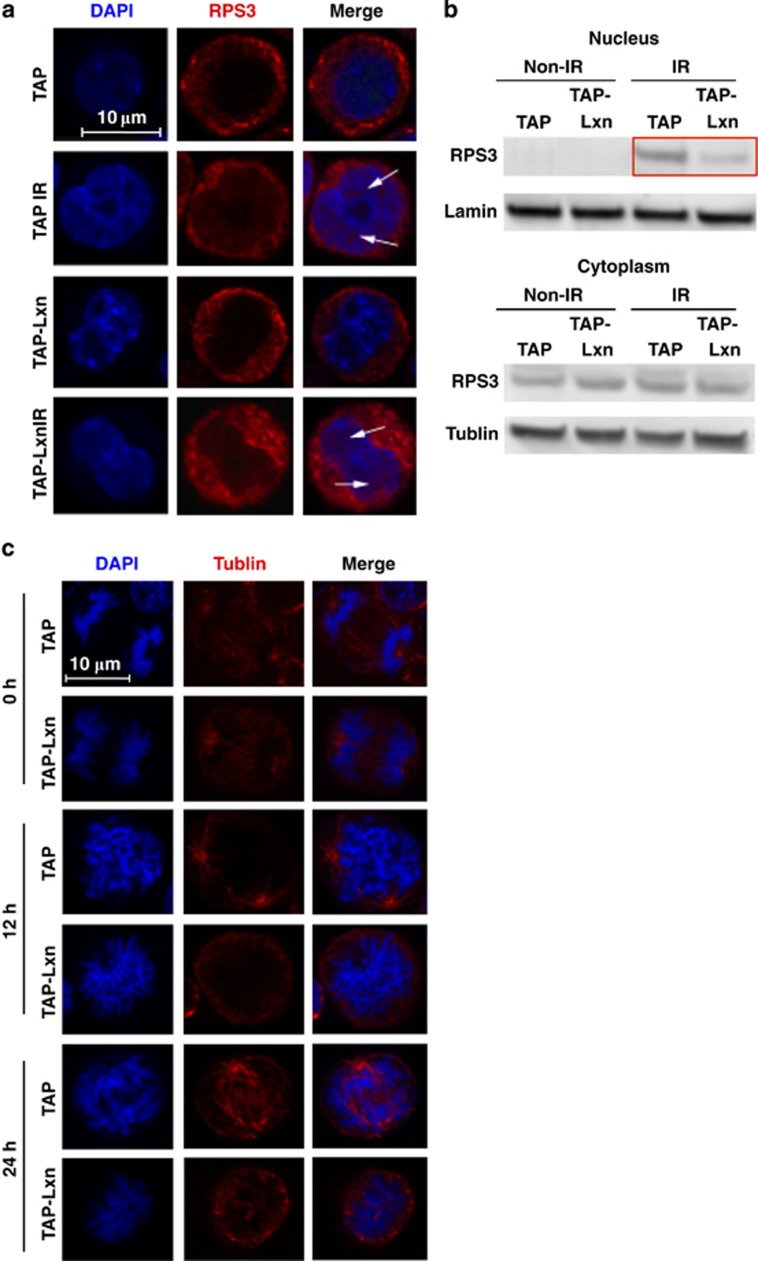
Lxn attenuates the radiation-induced nuclear translocation of Rps3. (**a**) Lxn reduces the radiation-induced nuclear translocation of Rps3. TAP-Lxn or TAP-transfected FDC-P1 cells were radiated with a dose of 6.5 Gy and Rps3 was immnunohistochemically stained with PE-conjugated Rps3 antibody before and 4 h post irradiation. Rps3 was detected in the nucleus only after radiation in both types of cells. (**b**) Lxn attenuates the nuclear translocation of Rps3. The nuclear and cytoplasm protein of TAP-Lxn or TAP-transfected FDC-P1 cells was extracted. Western blot was performed with Rps3 antibody, and the lamin and actin were used as nuclear and cytoplasm controls, respectively. (**c**) Lxn interferes mitotic spindle formation. Mitotic spindle and chromosome were immunohistochemically stained with *α*-tublin and DAPI, respectively, at 12 and 24 h post irradiation, and the representative imaging is shown. Lxn-overexpressing cells could not form the normal spindle and thus the duplicated chromosome failed to segregate and the mitosis was blocked. Scale bar represents 10 *μ*m

Rps3 is located in the cytoplasm of FDC-P1 cells (see Figure 1f). It was reported that Rps3 translocates into the nucleus upon DNA damage stress;^35^ therefore, we asked whether or not the nuclear translocation of Rps3 happens in Lxn-overexpressing FDC-P1 cells and how the increased level of Lxn affects this process. Immunohistochemical staining of Rps3 at 4 h post 6.5 Gy irradiation shows that Rps3 was translocated to the nucleus in both TAP-Lxn and control cells (Figure 7a). This result was further confirmed by western blot of Rps3 in the nuclear and cytoplasm protein lysates of both types of cells (Figure 7b). More interestingly, the amount of translocated Rps3 was significantly less in TAP-Lxn cells than in control (Figure 7b, top panel with red highlight), suggesting that Lxn inhibits nuclear translocation of Rps3 upon irradiation. It was reported that Rps3 facilitates spindle formation and chromosome movement during mitosis.^36^ We thus hypothesized that the reduced amount of nuclear Rps3 might interfere with spindle formation. By using anti-tubulin antibody and DAPI to immunohistochemically stain spindle and chromosome at 12 and 24 h after 6.5 Gy irradiation, we found that TAP-Lxn cells failed to form the normal mitotic spindle and could not complete the metaphase and the anaphase (Figure 7c). This could explain the cell-cycle arrest in G2/M phases (see Figure 5) as well as the increased chromosomal abnormality (see Figure 4).

### Rps3 knockdown increases radiation sensitivity and phenocopies radiation response of Lxn-overexpressing cells

**Figure 8:**
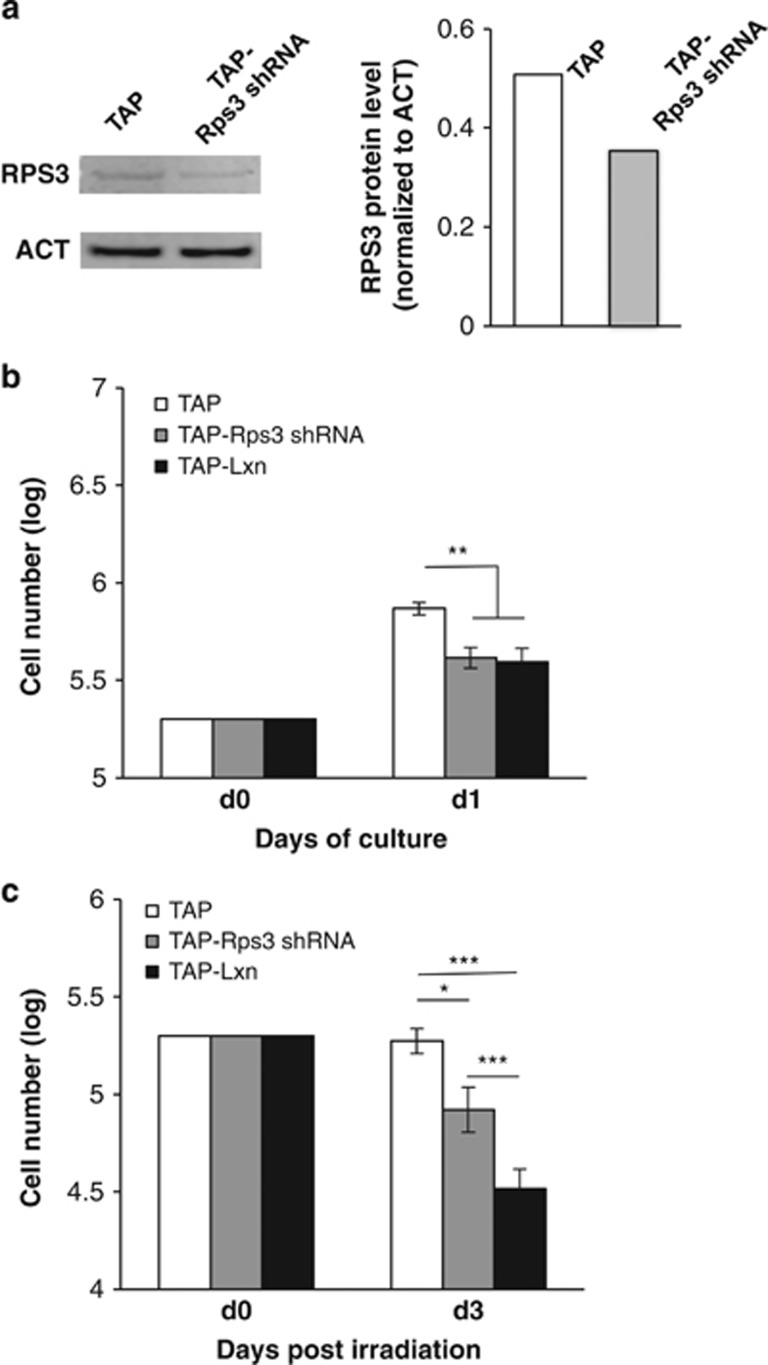
Rps3 knockdown increases radiation sensitivity and phenocopies radiation response of Lxn-overexpressing cells. (**a**) Knockdown Rps3 in FDC-P1 cells. FDC-P1 cells were transfected with Rps3 shRNA (TAP-Rps3 shRNA) or scramble shRNA (TAP). Rps3 protein level was detected by western blot (left panel) and quantified for knockdown efficiency (right panel). (**b**) Rps3 knockdown inhibits FDC-P1 growth and phenocopies the effect of Lxn overexpression on FDC-P1 cells (TAP-Lxn) in non-irradiation condition. (**c**) Rps3 knockdown increases the radiation sensitivity of FDC-P1 cells and produces a similar cytoxicity as Lxn overexpression. FDC-P1 cells with different treatments were subjected to 6.5 Gy irradiation and the cell growth was monitored for 3 days. The absolute numbers of FDC-P1 cells in different treatments were counted as previously described and represented as the average (± S.D.) out of three independent experiments. **P<0.01. ***P<0.001

To further confirm that role of Lxn in radiation response is mediated by inhibition of Rps3 activity, we knocked down Rps3 in FDC-P1 cells and examined its effect on cell growth and radiation response. It was reported that knocking down Rps3 protein by <50% would not adversely affect ribosomal instability and protein synthesis. The reason is that RNAi knockdown has a specific effect on cytosolic and ribosome-free Rps3, but not the ribosome-bound form.^20,22,25^ Therefore, we chose a shRNA sequence with ~33% knockdown of Rps3 in FDC-P1 cells (Figure 8a) and established a stable Rps3 knockdown cell line (TAP-Rps3 shRNA). The growth study shows that in comparison with control, the number of TAP-Rps3 shRNA cells was significantly decreased to a level similar to TAP-Lxn cells within 1 day of culture (*P*<0.01, Figure 8b), suggesting that Rps3 knockdown mimics the effect of Lxn overexpression. With 6.5 Gy irradiation, the growth of Rps3 knockdown cells was significantly inhibited compared with control although its number was higher than TAP-Lxn cells at day 3 post irradiation. This is probably due to the extent of Rps3 knockdown is not sufficient enough to suppress Rps3 translocation, thus radiation toxicity is not as significant as Lxn overexpression. These data suggest that RPS3 knockdown increases radiation sensitivity and phenocopies the effect of Lxn overexpression.

## Discussion

Lxn is a negative regulator of hematopoietic stem cell numbers.^3,30^ Recent reports have demonstrated that Lxn expression is absent or downregulated in several types of cancers and that Lxn overexpression inhibits tumor growth, suggesting that it may function as a tumor suppressor.^4, 5, 6, 7, 8,11^ Caboxypeptidase A is the only known Lxn binding protein, but we previously showed that the tumor suppressor activity of Lxn is not mediated *via* this canonical pathway.^5,10,14^ In this study, we examined the role of Lxn in myeloid leukemia, elucidated the underlying molecular mechanism, and evaluated its therapeutic potential.

Here, for the first time, we identify Rps3 as a novel Lxn binding protein. Rps3 is a component of the 40S ribosomal subunit and participates in ribosome biogenesis and protein synthesis. Recent reports have revealed its important extra-ribosomal functions, including DNA damage and repair,^29,37^ induction of apoptosis,^23^ regulation of mitosis,^36^ and involvement in the MDM2-p53 and NF-κB signaling pathways.^20,38^ Some of these biological functions are involved in the nuclear translocation of Rps3. However, the precise role of nuclear Rps3 in DNA damage and response is not yet clear. It can either facility repair due to its endonuclease activity^23^ or block the access of repairing machinery at sites of DNA damage, thus inhibiting repair.^21^ Nuclear Rps3 was also reported to associate with the mitotic spindle and regulate mitosis.^36^ In this study, we found that Lxn and Rps3 are located in the cytoplasm and interact with each other in the unstressed condition. With radiation-induced genotoxic stress, Rsp3 translocates into the nucleus, a result consistent with the previous report.^21,35^ Surprisingly, Lxn attenuates this process, resulting in less Rps3 moving to the nucleus upon irradiation. In addition, Lxn-overexpressing cell demonstrated abnormal mitotic spindle formation. Taken all these evidence together, we propose a model for the mechanism of action of Lxn in radiation response: Lxn sequesters Rps3 in the cytoplasm, resulting in less Rps3 translocation into the nucleus upon irradiation. Reduced level of nuclear Rps3 interferes with formation of the mitotic spindle, induces mitotic abnormalities and blocks cell-cycle progression.

To further address the role of Lxn-Rps3 interaction in cellular response to radiation, it would be necessary to manipulate Rps3 expression and examine its effect on radiation response. Since our data suggest that Lxn inhibits Rps3 activity (nuclear translocation), we first overexpressed Rps3 in Lxn-overexpressing FDC-P1 cells and found that high level of Rps3 severely suppressed cell growth, which precluded us from further assessing their radiation sensitivity (data not shown). This result is consistent with the previous report in which overexpression of Rps3 can induce apoptosis.^21^ Next, we employed knockdown of Rps3 in FDC-P1 cells to determine whether it can phenocopy Lxn-overexpressing cells. We found that >60% knockdown efficiency also produced cytotoxicity, which is consistent with the literature (data not shown).^20,22,25^ These findings are not surprising considering the important function of Rps3 in ribosomal assemble and protein synthesis. Therefore, we finally choose a Rps3 shRNA sequence that can knockdown Rps3 expression by 33% without direct cytotoxicity. We found that knockdown Rps3 can increase the radiation sensitivity and phenocopy Lxn-overexpressing cells, suggesting that Rps3 might participate in the Lxn-mediated cellular response to radiation. It should be admitted that the ideal strategy to address this question is to block the binding of Lxn and Rps3 and test its functional effect. One of the future studies will be focused on determining their binding domains, designing mutants that interrupt the interaction, and examining their functional effects.

Ectopic expression of Lxn suppresses FDC-P1 growth *in vitro*, suggesting its potential tumor suppressor function in myeloid leukemia. More importantly, we, for the first time, revealed that Lxn sensitizes leukemic cells to radiation and chemo agent-induced cytotoxicity in a dose-dependent manner, and at a certain dose it could completely eliminate cancer cells. Radiation or chemo agents induce DNA damage, and in normal cells, the DNA damage response primarily relies on the ATM-Chk2 signaling, and secondarily on the ATR-Chk1 pathway.^39^ Activation of these pathways will arrest normal cells in G1 *via* p53-dependent mechanism to allow time for DNA repair or they proceed to cell death if the damage cannot be repaired.^40^ In contrast, many cancers rely heavily on ATR-Chk1 mediated cell-cycle arrest in the S and G2 phases especially if cancers are deficient in p53.^41^ In consistence with these reports, our result showed that FDC-P1 cell is absent in p53 protein, thus its radiation response is specifically dependent on ATR-Chk1 signaling but not on ATM-Chk2 pathway. Very interestingly, Lxn inhibits the expression of ATR and phosphorylated Chk1 proteins in non-stress condition, resulting in accumulation of aberrant chromosomal structures in Lxn-overexpressing cells. Upon radiation, activation of ATR-Chk1 was further suppressed in Lxn-overexpressing cells, leading to the abrogation of S/G2 checkpoints and M-phase arrest. Because of the disturbed mitotic spindle, Lxn-overexpressing cells cannot pass through the M phase and ultimately undergo mitotic catastrophe and cell death. p53 mutations occur in approximately 50% of cancers, which make cancer cells less responsive to the genotoxic agents.^42^ Indeed, leukemia patients with p53 deficiency represent a group with a high resistance to radio- and chemotherapy and demonstrate the most aggressive disease courses.^43^ Therefore, activation of Lxn could sensitize p53-deficient tumor cells to radiation and chemotherapeutic agent, and potentiate their cytotoxicity, thus enhancing the efficacy of cancer treatment and reducing the relapse. It was reported that Lxn can be induced by clinically used retinoic acid^44^ and demethylating agent,^5^ suggesting the potential pharmaceutical methods to increase the abundance of Lxn in cancer patients.

Cancer is generally considered to originate from cancer stem cells, which are more resistant to anti-cancer therapies, leading to cancer relapse.^45^ Considering the regulatory role of Lxn in hematopoietic stem cells, we hypothesize that Lxn can be a promising molecular target, in combination with conventional anti-cancer therapy, to improve treatment efficacy and perhaps eradicate cancer stem cells. Our ongoing study focuses on its combinatorial role with chemotherapy in the treatment of leukemia and lymphoma as well as solid tumors.

## Materials and Methods

### Cell culture

Phoenix GP, 293TN and NIH 3T3 cell lines were cultured in Dulbecco's Modified Eagle's Medium (DMEM, Gibco, Grand Island, NY, USA) supplemented with 10% fetal bovine serum (FBS), 80 U/ml penicillin, and 80 mg/ml streptomycin. FDC-P1 cell line was cultured in DMEM supplemented with 25% mouse interleukin-3 (IL-3, BD Biosciences, San Jose, CA, USA) and 10% FBS, 80 U/ml penicillin and 80 mg/ml streptomycin. These cell lines were incubated in a humidified atmosphere of 5% CO_2_ in air at 37 °C. All cell lines used in this study were purchased from American Type Culture Collection (ATCC).

### Construction of overexpression and knockdown vectors and generation of cell lines

The retroviral vector Sf*β* 91 served as a control and the backbone for Lxn overexpression. It contained a 5' long terminal repeat (LTR) derived from myeloproliferative sarcoma virus (MPSV) and a 3' LTR derived from spleen focus forming virus (SFFV). The internal ribosomal entry site sequence derived from the encephalomyocarditis virus was used for simultaneous translation of the gene insert and enhanced green fluorescent protein (GFP) gene. Full-length mouse Lxn was fused in-frame to a N-terminal TAP tag and subcloned into the *Not*I site of retroviral vector sf*β* to create sf*β*-TAP-Lxn. A plasmid comprising only the TAP tag was used as a control. All of the cloned PCR products and constructed plasmids were sequenced. Phoenix GP packaging cells were transfected to produce retrovirus according to the previously described. FDC-P1 cells were then transfected by the retroviral supernatants of TAP control and sf*β*-TAP-Lxn vectors in the presence of 8 *μ*g/ml polybrene (Sigma-Aldrich, St. Louis, MO, USA). GFP+ cells were sorted by flow cytometry to create the stable cell line. For Rps3 knockdown, the Rps3 MISSION shRNA plasmid DNA (TRCN0000104309) and the scramble control plasmid DNA (pLKO.1-puro) were purchased from Sigma-Aldrich. The Rps3 shRNA sequence is 5′-CCGGCAACCAGGACAGAAATCATTACTCGAGTAATGATTTCTGTCCTGGTTGTTTTTG-3′. In all, 5 × 10^5^ FDC-P1 cells were infected with lentiviral particles carrying Rps3 shRNA or control sequence in the presence of 8 *μ*g/ml polybrene. After 48 h, puromycin (2 *μ*g/ml) was added into the cell culture for the selection of successfully infected cells. After 3 days of selection, survived cells were collected for the measurement of Rps3 protein level and growth and radiation study.

### TAP purification and MS

Whole-cell extracts were prepared from TAP-Lxn and TAP expressing FDC-P1 cells and purified using the InterPlay Mammalian TAP purification kit (Stratagene, Agilent Technologies, Santa Clara, CA, USA) following the manufacturer's protocol. Eluted proteins were separated on a 4–12% SDS-PAGE gel and visualized by SYPRO-RUBY staining. The differentially expressed-bands were cut into slices and processed for mass spectrometric (MC) analysis. The MC analysis was performed at the University of Kentucky, Center for Structural Biology Protein Core Facility and results were submitted to MASCOT for a database sequence similarity search.

### Co-immunoprecipitation analysis

FDC-P1 cells expressing Lxn were harvested and lysed on ice using TNTG lysis buffer (30 mM Tris-HCl, 150 mM NaCl, 1% Triton X-100 and 10% glycerol) with a protease inhibitor cocktail (Roche, Indianapolis, IN, USA) and 1 mM PMSF (Sigma-Aldrich). After centrifuging, the lysates were saved and used for immunoprecipitation. Briefly, 5 *μ*g of anti-Rps3 antibody or control normal rabbit IgG (Santa Cruz, Dallas, TX, USA) was incubated with cell extracts overnight at 4 °C, and subsequently collected by incubating with protein A/G-agarose beads for 1 h at 4 °C with rotation. The precipitates were washed five times with lysis buffer and then subjected to SDS-PAGE and detected by western blotting with chicken polyclonal anti-Lxn antibodies (Abcam, Cambridge, MA, USA). The same procedure was performed in a reciprocal manner in which chicken polyclonal anti-Lxn antibody (Abcam) was used to precipitate binding proteins and Rps3 was blotted by anti-Rps3 antibody.

### Immunofluorescence

Immunofluorescence was performed for detecting the localization of Lxn and Rps3 as well as for staining *γ*-H2A.X and spindle. For Lxn and Rps3 staining, the adsorbed FDC-P1 cells were incubated at 4 °C overnight with PBS containing 5% (v/v) normal goat serum, 0.1% (v/v) Triton X-100 and polyclonal antibodies against Lxn and Rps3 (diluted 1 : 500). After treatment with FITC and rhodamine-conjugated secondary antibody (diluted1 : 500) at 23 °C for 2 h, cells were stained with *γ*-H2A.X immunofluorescence staining was performed following the similar protocol. Cells were fixed at 0.5 h post 6.5 Gy irradiation and then incubated with the PE-conjugated antibody p-histone H2AX (S139) (Cell Signaling, Danvers, MA, USA) at 37 °C for 1 h. After washing three times, cells were stained with DAPI and the number of *γ*-h2A.X foci was counted. In all, 60–80 cells were counted per group for each experimental treatment. Data were expressed as the average number of *γ*-H2A.x foci per cells for each time point. The α-tublin antibody (Santa Cruz Biotechnology, Dallas, TX, USA) was used for spindle staining following a similar protocol. DAPI fluorescence signals were either taken with a Zeiss Axiovert-200 microscope using a high-resolution Zeiss digital camera (Carl Zeiss Inc., Jena, Germany) or taken with a FV1000 confocal microscope (Olympus, Center Valley, PA, USA).

### Measurement of cell growth

FDC-P1 cells transfected with TAP-Lxn and TAP control vectors were treated with (1) 3 Gy or 6.5 Gy *γ*-irradiation or (2) VP-16 (Etoposide, Sigma-Aldrich) at the concentration of 20 or 30 *μ*g/ml for 1 h. In each treatment group, 5 × 10^4^ cells were seeded into 24-well tissue culture plates (BD Falcon, Franklin Lakes, NJ, USA) and at day 0. The cell numbers were subsequently counted on a hemacytometer using trypan blue dye exclusion at different time points for 9 days. Fresh medium was added at each time point. FACS analysis was performed at each time point to measure the percentage of GFP+ cells.

### Chromosome stability analysis

Cells in log phase were cultured in fresh media with colcemid (5 *μ*l/ml of media, stock concentration 10 *μ*g/ml, Invitrogen, Life Technologies, Grand Island, NY, USA; Cat. No. 15210-040) for 2 h before harvest. Chromosome preparation was made according to the standard air drying procedure. The cells were harvested by trypsinization, washed with pre-warmed PBS twice, hypotonically treated (0.56% KCl, 20 min at 37 °C) and subsequently fixed in freshly prepared acetic acid-methanol (1 : 3). At least three changes were given in fixative before the cell suspension was dropped on to a pre-cleaned chilled microscopic glass slide and dried at room temperature at least for 1 day before staining. Giemsa staining method was applied for scoring metaphase chromosomes. Structural chromosome aberrations such as breaks, chromatid-type exchange (CTE), sister chromatid union (SCU), acentric fragments (acentrics), double minutes (Dimn), dicentric and ring chromosomes were scored under × 63 magnification. In each datum point, >150–340 metaphase spreads were scored. The modal chromosome number of FDC-P1 cell was 33 with 7 metacentric chromosomes. Considering the modal chromosome number 33, total number of chromosome investigated was calculated by multiplying modal chromosome number with number of metaphase spread scored for each datum point. Induction of total structural aberration between two groups (assuming 33 × number of metaphase spreads scored for the purpose of determining proportion values) was compared using *Z*-test to find the statistical significance between TAP control and TAP-Lxn cells. Considering one-sided test, if the Z-value is >1.64 or 2.33, then the difference in induction of structural translocations was considered as statistically significant at 5% level (*P*<0.05) or 1% level (*P*<0.01), respectively.^46^

### Flow-cytometry analysis

*Cell-cycle analysis*: BrdU staining was conducted using the BrdU Flow Kit (BD Bioscience, San Jose, CA, USA) following the manufacturer's instruction. In brief, 10^6^ cells were incubated with BrdU in the culture medium at the final concentration of 10 *μ*M for 1 h and cells were then fixed in 100 *μ*lBD Cytofix/Cytoperm buffer (BD Bioscience) at RT for 20 min. After washing three times with BD Perm/Wash buffer (BD Bioscience), DNase was added into the resuspended cells at the final concentration of 300 *μ*g/ml at 37 °C for 1 h. After washing, cells were incubated with PE conjugated anti-BrdU antibody (eBioscience, San Diego, CA, USA) at RT for 20 min. 7AAD (BD Bioscience) was added before flow-cytometry analysis. *Apoptosis and necrosis analysis*: To determine the profile of cell death after *γ*-irradiation, Annexin V staining was conducted at 0, 24, 48, 72 and 96 h after 6.5 Gy *γ*-irradiation. Annexin V-PE antibody (BD Bioscience) was used to stain the fixed cells following the manufacturer's protocol. 7AAD was added before flow-cytometry analysis. All flow-cytometry analyses were performed on a FACSAria II (Becton Dickinson Immunocytometry Systems, San Jose, CA, USA).

### Western blot

Total protein was extracted following the method of previous study. Cytoplasma and nucleus proteins were extracted separately by the Minute Cytoplasmic and Nuclear Extraction Kit (Invent Biotechnologies, Eden Prairie, MN, USA) following the manufacturer's protocol. For western blot, protein lysates were thawed and mixed with running buffer and a reducing agent (Novex, Life Technologies) according to the manufacturer's instructions and heated at 95 °C for 5 min. Samples were then separated in denaturing PAGE (Novex, 10% bis-Tris gel) using the equivalent of 4 × 10^5^ cells per lane at 185 V for 1 h. Following electrophoresis, samples were then transferred onto a PVDF membrane (Millipore, Billerica, MA, USA) by electro-blotting at 30 V for 1 h, which were subsequently blocked in 5% skimmed milk and probed with anti-p-Chk1 (S317) rabbit antibody (Cell Signaling), anti-Chk1 mouse antibody (Santa Cruz), anti-p-Chk2 (Thr68) rabbit antibody (Santa Cruz), anti-Chk2 rabbit antibody (Santa Cruz), anti-ATM rabbit antibody (Santa Cruz), anti-ATR rabbit antibody (Cell Signaling), anti-*β*-actin mouse antibody (Sigma-Aldrich), chicken polyclonal anti-Lxn antibodies (Abcam), anti-P53 mouse antibody (Santa Cruz), anti-laminB goat antibody (Santa Cruz), anti-caspase-3 rabbit antibody (Cell Signaling) and anti-ribosome protein S3 (Rps3) antibody (Santa Cruz), respectively. Primary antibodies were detected using alkalinephosphatase-conjugated secondary antibodies (Santa Cruz Biotechnology) and electro-chemi-fluorescent (ECF) reagent (Amersham Pharmacia Biotech, Piscataway, NJ, USA) according to the manufacturer's instructions. Blots were then visualized using a Molecular Dynamics STORM 860 system (GE Healthcare Life Sciences, Pittsburgh, PA, USA).

### Statistical analysis

Data were examined for homogeneity of variances (F-test), then analyzed by Student's *t*-test or one-way ANOVA using Tukey's test. Differences were considered as significant at *P*<0.05. All statistical analyses were conducted with a statistical package (SPSS 16.0 for Windows, IBM SPSS Statistics, IBM, Armonk, NY, USA).

## Abbreviations

Lxn: latexin
Rps3: ribosomal protein subunit 3
NCI: National Cancer Institute
TNF: tumor necrosis factor
Bcl-2: B-cell CLL/lymphoma 2
CPA: carboxypeptidase A
DNA: deoxyribonucleic acid
CPA4: carboxypeptidase A4
CPA1: carboxypeptidase A1
TAP: tandem affinity purification
MS: mass spectrometry
GFP: green fluorescent protein
kDa: kilodalton
LC–MS: liquid chromatography–mass spectrometry
p53: tumor protein p53
NF-κB: nuclear factor kappa-light-chain-enhancer of activated B cells
Co-IP: complex immunoprecipitation
Gy: gray (unit)
VP-16: etoposide phosphate
DSB: DNA double strand break
γ-H2A.X: phosphorylated H2A histone family, member X
ATM: ataxia telangiectasia mutated
ATR: ataxia telangiectasia and Rad3 related
Chk2: checkpoint kinase 2
Chk1: checkpoint kinase 1
pChk1: phosphorylated checkpoint kinase 1
pChk2: phosphorylated checkpoint kinase 2
BrdU: Bromodeoxyuridine
7AAD: 7-aminoactinomycin D
G0 phase: gap0/resting phase
G1 phase: growth 1/gap 1 phase
S phase: synthesis phase
G2 phase: pre-mitotic phase
M phase: mitotic phase
DAPI: 4',6-diamidino-2-phenylindole
RNAi: RNA interference
shRNA: short hairpin RNA
MDM2: mouse double minute 2 homolog
FDC-P1: factor-dependent cell progenitors
DMEM: Dulbecco's Modified Eagle's Medium
FBS: fetal bovine serum
IL-3: interleukin-3
ATCC: American Type Culture Collection
LTR: long terminal repeat
MPSV: myeloproliferative sarcoma virus
SFFV: spleen focus-forming virus
PCR: polymerase chain reaction
Tris-HCl: tris(hydroxymethyl)aminomethane hydrochloride
NaCl: sodium chloride
PMSF: phenylmethylsulfonyl fluoride
SDS-PAGE: sodium dodecyl sulfate polyacrylamide gel electrophoresis
PBS: phosphate-buffered saline
FITC: fluorescein isothiocyanate
KCl: potassium chloride
CTE: chromatid-type exchange
SCU: sister chromatid union
Dimn: double minutes
FACS: fluorescence-activated cell sorting
PAGE: polyacrylamide gel electrophoresis
PVDF: polyvinylidene fluoride
WB: western blot
