# Supplementary material for: Latexin sensitizes leukemogenic cells to gamma-irradiation-induced cell-cycle arrest and cell death through Rps3 pathway
Source: Cell Death Dis. 2014 Oct 23;5(10):e1493–. doi: 10.1038/cddis.2014.443 (PMC4237263; doi:10.1038/cddis.2014.443)
Supplement: Supplementary Table S1 [file cddis2014443x1.pdf]

Table S1. Potential Latexin binding proteins identified by Mass spectrometric analysis

Name	Score
Latexin	242
Ribosomal protein S3	118
Glial fibrillary acidic protein	77
Acidic ribosomal protein p0	128
Tublin, beta chain	462
H+transporting 2-sector ATPase alpha chain	458
Eukaryotic translation factor 1 alpha 1	424
Tublin alpha chain	242
HSP8	609
HSP84	387
HSP86	297

Whole cell extracts were prepared from TAP-Lxn and TAP expressing FDC-P1 cells and purified using the InterPlay Mammalian TAP purification kit (Stratagenel. Eluted proteins were separated on a 4%-12% SDS PAGE gel and visualized by SYPRO-RUBY staining. The differentially expressed-bands were cut into slices and processed for LC-mass spectrometric (MC) analysis. The LC-MC analysis was performed at the University of Kentucky, Center for Structural Biology Protein Core Facility and results were submitted to MASCOT for a database sequence similarity search. Eleven protein candidates were identified to be prominent hits.
